# Erythema Multiforme-Like Lesions Revealing Systemic Lupus Erythematosus

**DOI:** 10.7759/cureus.86574

**Published:** 2025-06-23

**Authors:** Hanae Tassine, Abire Allaoui, Abdelhamid Naitlhou

**Affiliations:** 1 Department of Internal Medicine, Mohammed VI International University Hospital, Mohammed VI University of Health Sciences (UM6SS), Casablanca, MAR; 2 Department of Internal Medicine, Cheikh Khalifa University Hospital, Mohammed VI University of Health Sciences (UM6SS), Casablanca, MAR

**Keywords:** cutaneous lupus erythematosus, erythema multiforme, erythema multiforme-like lesions, rowell syndrome, systemic lupus erythematosus

## Abstract

Rowell syndrome is a rare clinical entity, defined by the association of systemic lupus erythematosus (SLE) and erythema multiforme (EM)-like skin lesions, accompanied by distinctive immunological findings such as a speckled antinuclear antibody (ANA) pattern. It predominantly affects women and requires specific diagnostic criteria. Management depends on the extent and severity of organ involvement. We report the case of a 69-year-old woman who developed severe mucocutaneous erythema multiforme-like lesions and systemic symptoms. Clinical and laboratory findings supported the diagnosis of SLE. Targeted treatment combining corticosteroids and hydroxychloroquine resulted in marked clinical and biological improvement.

## Introduction

Systemic lupus erythematosus (SLE) is a complex autoimmune disease that can present with a variety of dermatological manifestations. These lesions often provide important diagnostic clues. Rowell syndrome, first described by Rowell et al. in 1963, is a rare entity characterized by the combination of lupus erythematosus and erythema multiforme (EM)-like lesions with a characteristic immunological pattern that includes a positive rheumatoid factor, speckled antinuclear antibodies (ANA), positive anti-Ro/SSA antibodies, and, positive anti-La/SSB antibodies [1]. Though rare, Rowell syndrome (RS) remains a subject of ongoing debate, with some considering it a distinct clinical entity and others viewing it as part of the broader lupus spectrum or a coincidental overlap with EM [2,3]. Nevertheless, recognizing this presentation is clinically important, as EM-like lesions in the context of lupus may indicate active systemic disease and help guide therapeutic decisions. Treatment generally involves systemic corticosteroids, although some patients may require additional immunosuppressive therapy [3,4]. We report a case of RS in which EM-like lesions were the initial clue to an underlying diagnosis of SLE, highlighting the diagnostic challenges and clinical significance of this unusual cutaneous presentation.

## Case presentation

We report the case of a 69-year-old female patient with a recent diagnosis of vitamin B12 deficiency anemia, who had received treatment months prior to hospitalization, resulting in hematological recovery. Two weeks prior to hospitalization, she developed gingivostomatitis accompanied by diffuse cutaneous lesions and painful ulcerations of the oral and genital mucosa. These symptoms were associated with asthenia, anorexia, unquantified weight loss, and a persistent fever of 38.5 °C. There was no history of recent drug exposure commonly associated with erythema multiforme or Stevens-Johnson syndrome, apart from vitamin B12 supplementation initiated several months earlier.

**Figure 1:**
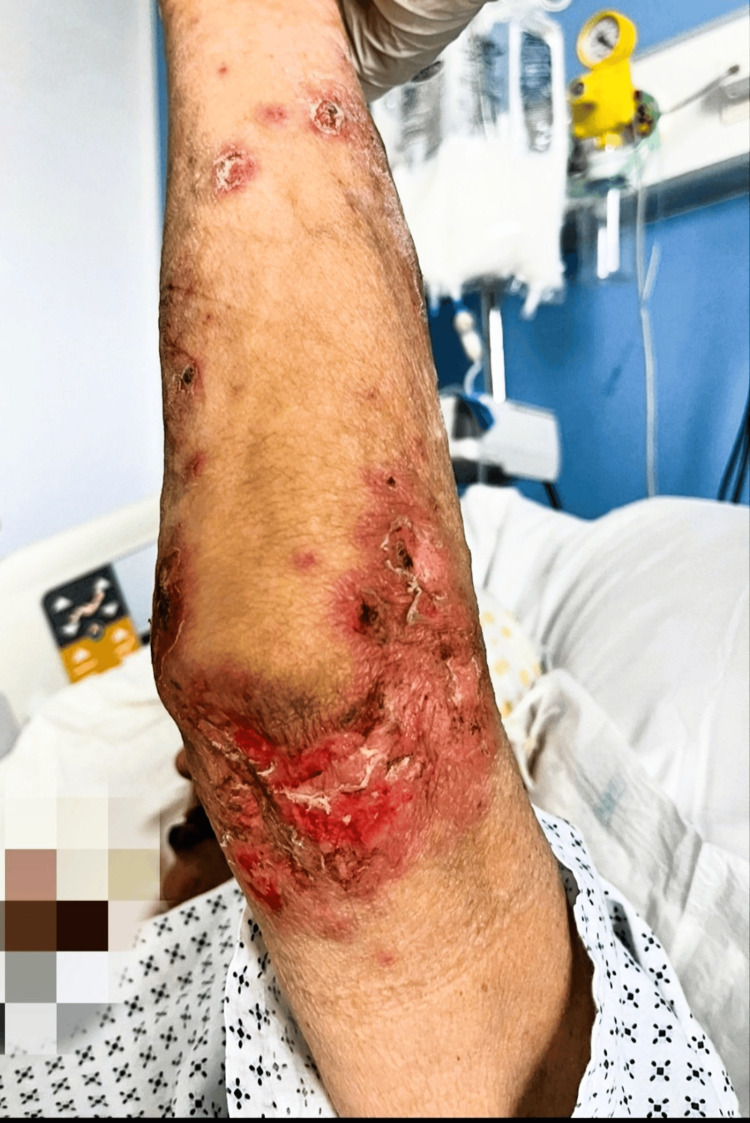
Multiple erythematous to violaceous lesions with overlying scale, crust, and ulceration on the extensor surface of the arm.

**Figure 2:**
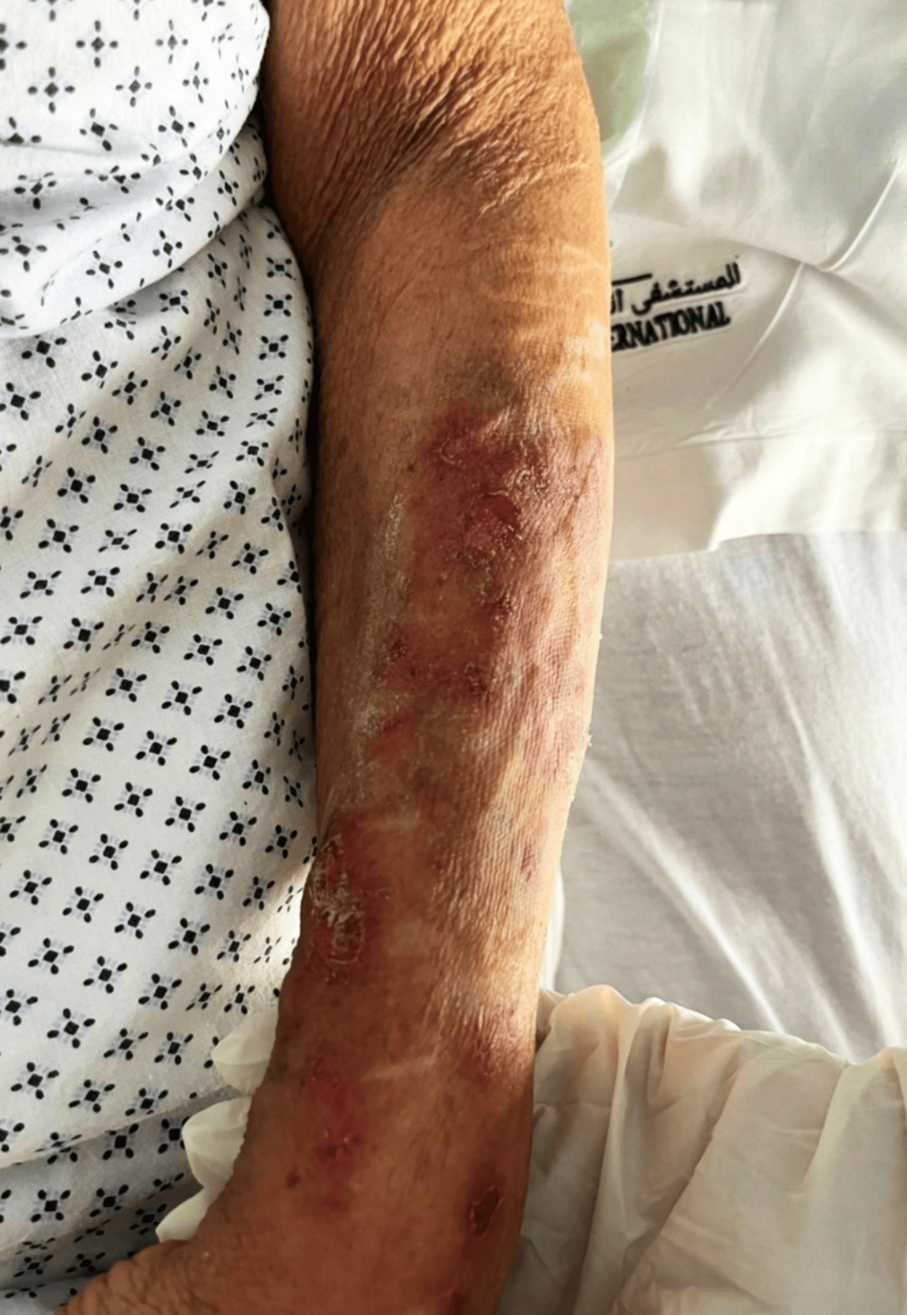
Multiple erythematous plaques on the anterior surface of the forearm.

**Figure 3:**
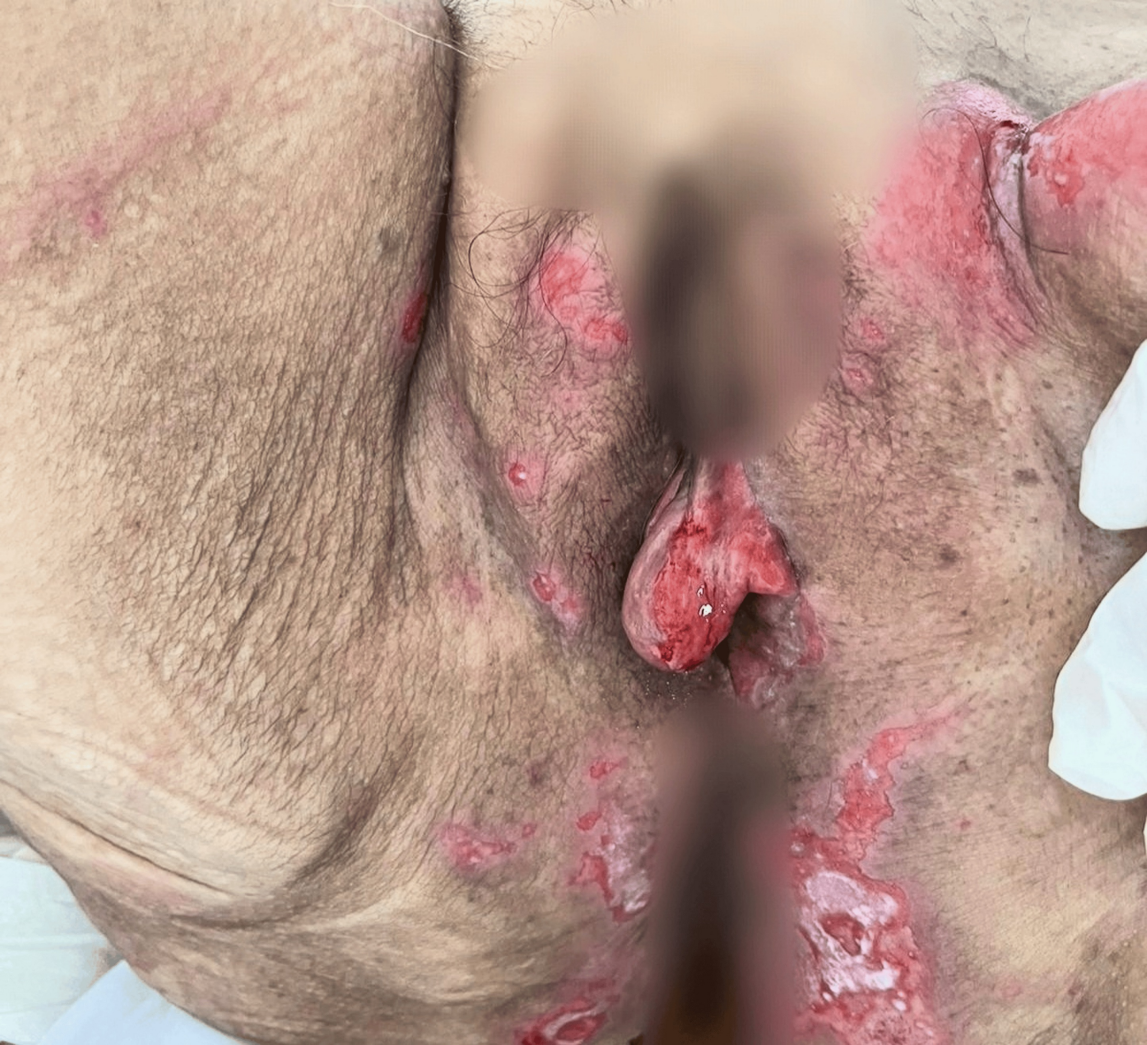
Multiple erythematous lesions interspersed with superficial ulcerations, involving the vulva, perineum, and inguinal folds.

Clinical examination revealed hemorrhagic oral lesions covered with crusts and bleeding upon contact, as well as widespread erythematous plaques (Figures 1-2), some target-shaped, with overlying bullous lesions. These were located on the face, back, arms, lower limbs, and palms. In addition, flame-shaped lesions were observed on the fingers, and diffuse erythematous plaques were noted on the scalp as well as the genital area (Figure 3). Although targetoid lesions were noted on clinical examination, these specific lesions were unfortunately not documented in the available photographs. A dermatology consultation was performed and confirmed erythema multiforme-like lesions. Mucosal cultures were not performed, as they were declined due to bleeding upon minimal contact.

Laboratory investigations revealed significant findings, including normochromic, normocytic anemia with hemoglobin at 9.1 g/dL, lymphopenia, and moderate renal impairment. Inflammatory markers were elevated, with erythrocyte sedimentation rate (ESR) at 61 mm and C-reactive protein (CRP) at 51.5 mg/L, along with markedly elevated ferritin. Immunological tests were strongly positive for multiple antibodies, including ANA at a titer of 1:1280, anti-dsDNA, anti-SSA (Ro), anti-SSB (La), and others consistent with SLE. Serological tests for cytomegalovirus (CMV) and herpes simplex virus (HSV) showed positive IgM and IgG antibodies, though PCR testing was negative. As the patient was symptomatic with complaints of pollakiuria and dysuria, a urine culture was performed, and *Klebsiella pneumoniae* was isolated. An initial urinalysis revealed hematuria and leukocyturia, but these findings were attributed to the urinary tract infection. A repeat urinalysis performed after resolution of the infection showed no hematuria or leukocyturia, and 24-hour proteinuria remained within normal limits at 0.14 g/24 h, suggesting no active renal involvement. The full laboratory findings are summarized in Table 1. Histopathological examination of the skin biopsy demonstrated focal keratinocyte necrosis associated with a relatively extensive dermoepidermal detachment. The underlying dermis revealed a mild lymphoplasmacytic infiltrate with perivascular and periadnexal distribution. However, direct immunofluorescence was not performed in this case.

**Table 1:** All the laboratory investigations performed and their results. SGOT: serum glutamic-oxaloacetic transaminase; AST: aspartate aminotransferase; SGPT: Serum glutamic pyruvic transaminase; alanine transaminase; ESR: erythrocyte sedimentation rate; CRP: C-reactive protein; HSV: herpes simplex virus; CMV: cytomegalovirus; ANA: antinuclear antibody; SSA/SSB: Sjögren’s antibody testing; DSF: distal splicing factor; snRNP: small nuclear ribonucleoprotein

Category	Parameter	Result	Reference Range
Hematology	Hemoglobin	9.1 g/dL	12–16 g/dL (female)
	Lymphocyte count	620 cells/mm³	1,000–4,800 cells/mm³
Renal Function	Urea	1.21 g/L	0.15–0.45 g/L
	Creatinine	24.7 mg/L	6–12 mg/L (female)
	24-hour proteinuria	0.14 g/24 h	<0.15 g/24 h
Liver Function	SGOT/AST	25 units/L	<35 U/L
	SGPT/ALT	30 units/L	<35 U/L
Inflammatory Markers	ESR	61 mm	<20 mm/h (female)
	CRP	51.5 mg/L	<5 mg/L
	Ferritin	>2000 ng/mL	20–200 ng/mL (female)
Serology	Hepatitis B, C, HIV, syphilis	Negative	–
	Epstein-Barr virus	Negative	–
	Mycoplasma	Negative	–
	CMV IgG/IgM	Positive	–
	HSV IgG/IgM	Positive	–
	CMV PCR	Negative	–
	HSV PCR	Negative	–
Serum Protein Electrophoresis	Gamma globulins	Polyclonal hypergammaglobulinemia	–
	Alpha-1 globulins	Elevated	–
Immunology	Rheumatoid factor	Positive	–
	ANA	1:1280, homogeneous	–
	Perinuclear enhancement	Positive	–
	Anti-DNA antibodies	1:40	–
	Anti-nucleosome	Strongly positive	–
	Anti-histone	Strongly positive	–
	Anti-SSA (Ro)	Strongly positive	–
	Anti-SSB (La)	Strongly positive	–
	Anti-Sm	Positive	–
	Anti-DSF-70	Positive	–
	Anti-U1-snRNP	Low titer positive	–
	Anti-Ku	Low titer positive	–
	Anti-Mi-2a	Low titer positive	–
	Anti-PM-Scl 75	Low titer positive	–
	Anti-Ro52	Strongly positive	–
	Direct Coombs test	Positive	–

Therapeutic management included two components. Symptomatic treatment consisted of targeted antibiotic therapy for the urinary infection, parenteral nutrition due to oral feeding intolerance, and daily skin and mucosal care. Given the presence of gingivostomatitis and the positive HSV serology (both IgM and IgG), antiviral therapy with acyclovir was initiated despite a negative HSV PCR. This decision was made based on clinical suspicion, reinforced by the positive IgM result, and the need to initiate high-dose corticosteroid therapy, which could exacerbate an undiagnosed herpetic infection. Specific treatment for SLE included systemic corticosteroids at a dose of 1 mg/kg/day of prednisone, combined with hydroxychloroquine.

The outcome was marked by improvement in both clinical and biological parameters. After one month of treatment, the skin and mucosal lesions had completely resolved, and renal function normalized, indicating a favorable response with an 8-month follow-up.

## Discussion

RS is an uncommon condition marked by the association of SLE and erythema multiforme (EM)-like lesions, along with distinct immunologic abnormalities. First identified by Rowell et al. in 1963 [1], its classification as a distinct clinical entity remains debated. Nonetheless, numerous case reports and literature reviews have enhanced our understanding of its presentation, diagnostic criteria, pathogenesis, and management strategies [2,3,5].

Over the years, various diagnostic criteria have been proposed for RS. The most frequently cited are those proposed by Zeitouni et al. (2000) [6]. The major criteria include SLE, discoid lupus erythematosus (DLE), or subacute cutaneous lupus erythematosus (SCLE); EM-like lesions with or without mucosal involvement; and a positive ANA with a speckled pattern. The minor criteria include chilblains, anti-Ro or anti-La antibody, and a positive rheumatoid factor (RF). In order to make the diagnosis, all three major criteria and at least one minor criterion are required [6].

RS predominantly affects females, with a mean age of onset around 72.5 years [3]. However, cases in males and adolescents have also been reported [5]. Patients often present with erythema multiforme-like lesions, including targetoid macules and papules, which may involve mucosal surfaces such as the oral cavity and genitalia [7]. SLE may precede, coincide with, or follow the onset of EM-like lesions, complicating the clinical picture [7,8]. Neurological and psychiatric symptoms, including seizures and cognitive impairment, have also been associated with RS, emphasizing the importance of a thorough systemic evaluation [9]. In our case, the patient presented with mucosal involvement but with no evidence of internal organ manifestations. The erythema multiforme-like lesions were the initial presentation and ultimately led to the diagnosis of lupus, emphasizing the importance of considering RS in patients with atypical or unexplained EM-like eruptions.

The exact mechanisms of RS remain unclear. However, it is hypothesized that the presence of anti-Ro antibodies may trigger a type IV hypersensitivity reaction, leading to EM-like lesions in the context of LE [3].

The management of RS generally aligns with therapeutic strategies used for SLE, often involving systemic corticosteroids, antimalarial agents such as hydroxychloroquine, and, in more resistant cases, immunosuppressive drugs like azathioprine [3]. For patients who do not respond adequately to conventional treatments, alternative options, including rituximab, cyclosporine [4], and, more recently, biologic agents such as belimumab and anifrolumab, have demonstrated encouraging outcomes [10]. The patient in our case demonstrated a favorable response to a combination of hydroxychloroquine and systemic corticosteroids, with sustained clinical remission observed over an 8-month follow-up period. This remission was maintained despite the gradual tapering of corticosteroid therapy, and without the need for additional immunosuppressive agents, suggesting effective disease control with standard first-line treatment.

The prognosis of RS varies depending on the severity of the lesions, the extent of systemic involvement, and the response to treatment. Early recognition and tailored treatment are crucial in improving outcomes and preventing complications.

## Conclusions

This case highlights the diagnostic complexity of RS, a rare condition at the intersection of SLE and erythema multiforme-like lesions. Early recognition, supported by immunological profiling, is crucial for initiating timely immunosuppressive therapy. The favorable clinical response observed reinforces the importance of considering RS in similar mucocutaneous presentations to improve patient outcomes. Given the overlap with other mucocutaneous conditions such as SCLE, drug-induced erythema multiforme, and bullous lupus, careful clinical and immunological evaluation is essential to establish an accurate diagnosis, especially when histological data are limited. Continued documentation of case reports and comprehensive literature reviews is essential to enhance clinical awareness, refine diagnostic criteria, and develop effective treatment strategies.
